# Genome Assembly and Structural Variation Analysis of *Luffa acutangula* Provide Insights on Flowering Time and Ridge Development

**DOI:** 10.3390/plants13131828

**Published:** 2024-07-03

**Authors:** Aizheng Huang, Shuo Feng, Zhuole Ye, Ting Zhang, Shenglong Chen, Changming Chen, Shijun Chen

**Affiliations:** 1 Institute of Agricultural Science Research of Jiangmen, Jiangmen 529060, China; haz6242@163.com; 2 College of Horticulture, South China Agricultural University, Guangzhou 510642, China; fengshuo0929@gmail.com (S.F.);; 3 Dongguan Agricultural Scientific Research Center, Dongguan 523086, China

**Keywords:** *Luffa acutangula*, chromosome-level genome, structural variation, ridge development, flowering time

## Abstract

*Luffa* spp. is an important worldwide cultivated vegetable and medicinal plant from the Cucurbitaceae family. In this study, we report a high-quality chromosome-level genome of the high-generation inbred line SG261 of *Luffa acutangula.* The genomic sequence was determined by PacBio long reads, Hi-C sequencing reads, and 10× Genomics sequencing, with an assembly size of 739.82 Mb, contig N50 of 18.38 Mb, and scaffold N50 of 56.08 Mb. The genome of *L. acutangula SG261* was predicted to contain 27,312 protein-coding genes and 72.56% repetitive sequences, of which long terminal repeats (LTRs) were an important form of repetitive sequences, accounting for 67.84% of the genome. Phylogenetic analysis reveals that *L. acutangula* evolved later than *Luffa cylindrica*, and *Luffa* is closely related to *Momodica charantia*. Comparing the genome of *L. acutangula SG261* and *L. cylindrica* with PacBio data, 67,128 high-quality structural variations (SVs) and 55,978 presence-absence variations (PAVs) were identified in *SG261*, resulting in 2424 and 1094 genes with variation in the CDS region, respectively, and there are 287 identical genes affected by two different structural variation analyses. In addition, we found that the transcription factor FY (*FLOWERING LOCUS Y*) families had a large expansion in *L. acutangula SG261* (flowering in the morning) compared to *L. cylindrica* (flowering in the afternoon), which may result in the early flowering time in *L. acutangula SG261*. This study provides valuable reference for the breeding of and pan-genome research into *Luffa* species.

## 1. Introduction

*Luffa* spp. (2n = 26), belonging to the Cucurbitaceae family, includes nine species, namely *L. cylindrica* (*L. aegyptiaca*), *L. acutangula*, *L. quinquefilia*, *L. operculata*, *L. saccata*, *L. sepium*, *L. graveolens*, *L. echinata*, and *L. astorii* [1]. There are two domesticated types: the angled loofah (ridge gourd), *Luffa acutangula* L. Roxb., and the smooth loofah (smooth gourd), *Luffa cylindrica* L. Rome., which are annual and largely monoecious [2,3]. *Luffa* is native to India and widely cultivated in tropical and subtropical areas, such as China, Malaysia, India, Korea, Thailand, Central America, and Africa [4,5]. As a vegetable plant, it is rich in nutrition and contains many secondary metabolites which can be used as medicinal components, including saponins, alkaloids, flavonoids, anthraquinones, and steroids. Its isolated compounds possess broad pharmacological activities such as antidiabetic, hepatoprotective, antiulcer, anticancer, immunomodulatory, and antihyperlipidemic properties [6].

*L. acutangula* and *L. cylindrica* are two vegetable species commonly found in South and Southeast Asia. *L. acutangula* is widely grown; however, *L. cylindrica* is considered an underutilized crop [7]. There are obvious phenotypic differences between *L. cylindrica* and *L. acutangula*. There is a profound variation in fruit size, length, shape, and color [7]. The smooth gourd fruit is smooth and cylindrical, and the ridge gourd fruit has a tapering neck with some prominent longitudinal ridges [8]. In addition, the two species can also be distinguished according to color and flowering time: *L. cylindrica* has bright yellow flowers that bloom in the early morning (4–8 a.m.), while *L. acutangula* has pale cream flowers that bloom in the late afternoon (5–8 p.m.) [2,8]. It is very meaningful to study the difference in flowering time between these two species, which can contribute to exploring the different flowering mechanisms in these two species.

The process of introgressing desired traits acquired from interspecific hybridization into elite cultivars can be made more efficient using molecular breeding approaches. With the development of sequencing technology, genome assembly has also developed rapidly. The genome of *L. cylindrica* was sequenced and assembled for the first time using next-generation sequencing (NGS) technology (small insert (220 bp) library) in 2017, and a genome of 885.01 Mb was obtained [9]. Subsequently, three genome assemblies of *L. cylindrica* have been completed utilizing the PacBio long read single-molecule real-time (SMRT) sequencing platform [10,11,12], while only one genome assembly of *L. acutangula* has been completed [10].

To explore genetic variability and diversity that exist within a species, structural variation (SV) studies have been recently reported for some important crop plants, including corn, soybean, and rice [13]. Structural variations (SVs), including insertion, deletion, tandem repeat, inversion, translocation, copy number variations (CNVs) with a length of more than 50 bp, and chimerism variations present a more complicated situation [14]. Compared with single-nucleotide polymorphisms (SNPs), SVs occupy a larger proportion in the variation base and have significant impact on variations in the genome [15]. Numerous studies have indicated that SVs play a critical role in genome evolution and genetic control of agronomic traits such as flowering time, fruit size, and stress resistance [13,16] and gradually have become an increasingly important research field. Likewise, presence/absence variations (PAVs) also can contribute to trait variation [17].

The first attempt to perform a genome survey sequencing of *Luffa cylindrica* using next-generation sequencing (NGS) technology was carried out by An et al. [9]. Recently, Zhang et al. (2020) reported a de novo assembly of the L. cylindrica genome, utilizing the Pacific Biosciences (PacBio) sequencing platform [12]. Pootakham et al. previously assembled acutangula and cylindrica genomes and investigated alternative splicing events in *Luffa* [10], but little research has been conducted on the structural variability of *L. acutangula* and *L. cylindrica*. The narrow genetic and genomic resources obviously limited the breeding improvement of Luffa. In this study, we de novo assembled the genome of the high-generation inbred line *L. acutangula SG261* by combining the PacBio long reads, Hi-C data, and 10× Genomics short reads. Genome annotation, comparative genome analysis, and structure variation identification were conducted based on the assembled genome sequences. PacBio long-read sequencing data were used to identify SVs between *L. acutangula* and *L. cylindrica*. The present study provides a valuable genetic resource for deciphering the genome evolution of *Luffa* species and is positioned to serve as the reference genome guiding the molecular breeding of *Luffa* crops.

## 2. Materials and Methods

### 2.1. Plant Materials

**Figure 1:**
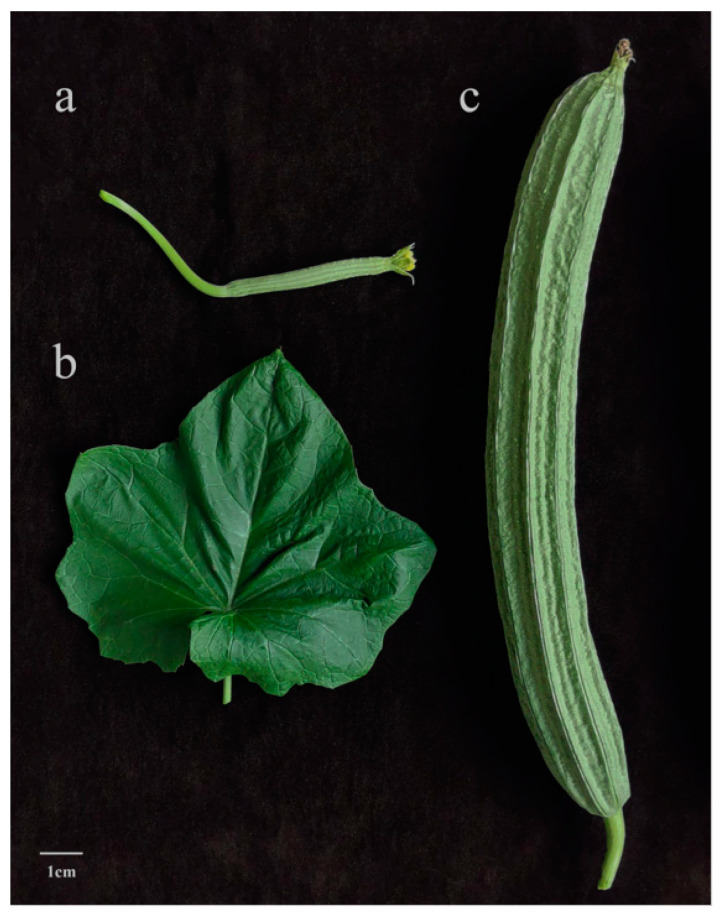
Morphological characteristics of the *L. acutangula* inbred line SG261: (**a**) young fruit; (**b**) leaf; (**c**) mature fruit.

The highly inbred line SG261 of *L. acutangula* was used in this study. This line has distinctive ridges and large flowers (blooming at night) (Figure 1). The seedlings were given adequate sunlight, nutrients, and water for normal growth in a greenhouse at Jiangmen Institute of Agricultural Sciences, in Jiangmen, Guangdong, China.

### 2.2. Section Observation

The ridges of young (the day after pollination) and mature fruits (eighth day after pollination) of *L. acutangula* and *L. cylindrica* were sampled to make paraffin sections by Henan Honglin Educational Instrument Co., Ltd. (Henan, China). After the material was taken, it was fixed, rinsed, dehydrated, permeabilized, embedded, sectioned, spread, baked, and dehydrated. Then, the sections were stained with plant tissue staining solution Safranin O-fast green and observed on an Olympus microscope [18].

### 2.3. Genome Sequencing

Young fresh leaves of SG261 were collected for genomic DNA extraction and sequencing. Total DNA was extracted using the cetyltrimethylammonium bromide (CTAB) method to construct PacBio and short-read libraries. The short-read libraries with an insertion size of 350 bp were prepared using VAHTS Universal DNA library preparation kit (Vazyme, Nanjing, China) by the Beijing Genomics Institute (BGI), and the insertion fragments of the library were detected using the Agilent Bioanalyzer 2100 (Agilent Technologies, Santa Clara, CA, USA). The library was sequenced on the MGI-SEQ 2000 sequencing platform to produce pair-end sequence data (2 × 150 bp) [19]. For the PacBio library, high-molecular-weight DNA was extracted as above. About 30 ug of high-molecular-weight DNA was used to prepare template library of 30–40 kb using the BluePippin Size Selection system (Sage Science, Beverly, MA, USA). The library was then prepared by adding specific splice sequences to both ends of the fragmented DNA. The library was sequenced on the PacBio Sequel II sequencing platform using standard sequencing for continuous long reads (CLRs) to generate long reads by SMRT sequencing chips [20].

To obtain Hi-C sequencing data, the chopped young leaves of SG61 were vacuum-fixed with 2% fresh formaldehyde in NIB buffer for 45 min, and glycine was added to a concentration of 0.375 M. The reaction was carried out for 5 min, and then liquid nitrogen was added and ground to powder. Then, it was filtered through a layer of Miracloth membrane. Isolated cells were lysed, and proteins were broken down using proteinase K at 65 degrees centigrade. DNA was purified by QIAamp DNA Mini Kit (Qiagen), and then dotted flat-end junctions were removed using Dynabeads^®^ MyOne™ Streptavidin C1 (Thermofisher). Then, sequencing library was prepared using the NEBNext^®^ Ultra™ II DNA library Prep Kit and sequenced on the BGI MGI-SEQ 2000 platform (San Diego, CA, USA) using the 150 PE mode.

### 2.4. RNA-Seq Library Construction and Sequencing

Fresh, disease-free, big flower buds before flowering were selected. The mixed flower buds from 5 individual plants were used for RNA extraction and transcriptome sequencing in three biological replicates. Total RNA was extracted using Trizol reagent (Invitrogen, Waltham, MA, USA), and the RNA purity and integrity were assessed by NanoDrop 2000 spectrophotometer (NanoDrop Technologies, Wilmington, DE, USA) and by Agilent 2100 Bioanalyzer (Agilent Technologies, CA, USA). After passing the quality inspection, the sequencing library was constructed using the VAHTS Universal V6 RNA-seq Library Kit for MGI (Vazyme, Nanjing, China) and quantified by Qubit 3.0 Fluorometer (Life Technologies, Carlsbad, CA, USA). The library was then assessed for size and quality by the Agilent 2100 Bioanalyzer (Agilent Technologies, CA, USA) and sequenced on the MGI-SEQ 2000 platform.

### 2.5. De Novo Sequencing and Genome Assembly

The raw BGI short reads were cleaned using SOAPnuke version 2.1.0 [21], with parameters “-lowQual = 20, -nRate = 0.005, -qualRate = 0.5”. The genome size and heterozygosity of SG261 were estimated by GCE version 1.0.2 [22] using k-mer frequency distribution generated from cleaned BGI short reads, with the following parameters: “-m 1 -D 8 -b 0 -H 1”. PacBio long reads were filtered (retaining the top 72 Gb longest reads, ~100 × genomic coverage) and then employed for correction using MECAT2 version 2.1 [23] and then trimmed and assembled using CANU version 2.0 [24], with parameters “GenomeSize = 879,830,000”. Bowtie2 version 2.4.5 [25] was used to map the 10× BGI data to the assembly, and Pilon version 1.23 software [26] was used to polish the assembly.

After assembling the genome contigs of SG261, Hi-C data were used to assign genome assembly to chromosome level. Hi-C clean reads were aligned onto the assembled genome by Juicer v1.5.7 [27], and then software 3D-DNA v180114 [28] was used to preliminarily cluster and orient the data. Then, the assembly genome was adjusted, reset, and aggregated by JuiceBox v1.11.08 [29] to improve the quality of chromosome assembly. The chromosome assembly genome was mapped to a published *L. acutangula* genome [10] using minimap2 v2.24 [30], and the final genome was obtained by reverse complementation and direction adjustment according to the mapped results.

To evaluate the assembly results, BWA-mem v0.7.17 [31] was used to map the Hi-C data to the assembled genome, and a Hi-C contact map was generated by HiCExplorer v3.5.3 software [32] with a 100 kb window and a threshold of −1.5 to 5. Finally, BUSCO v3.0.2 [33] was used to estimate the integrity of the assembly genome based on the Embryophyta_odb9 database (n = 1440).

### 2.6. Repetitive Element Annotation and Gene Prediction

Transposable elements (TEs) were annotated by EDTA v1.9.6 [34]. Taking the results of EDTA as a library, RepeatMasker v4.1.2.p1 [35] was used to annotate incomplete repetitive elements. Tandem Repeats Finder (TRF) v4.09 [36] was used to annotate the tandem repeats (TRs) with default parameters.

Three data sets from the RepeatMasker library (RMlib), expressed sequence tags (EST), and peptides were used for protein-coding gene prediction using the MAKER v3.31.10 pipeline [37]. To improve the quality of gene annotation, four cycles of annotation were conducted using MAKER. For expressed sequence tags, raw transcriptome data from flower buds of *L. acutangula SG261* were cleaned using SOAPnuke v2.1.0, and the clean data were mapped to the assembly genome by HISAT2 v2.2.1 [38]. StringTie v2.2.1 [39] was used to reconstruct genome annotations and integrate annotations using the merge function and finally extract the transcription tag by gffread v0.12.1 [40]. The peptides produced were predicted by TransDecoder v5.5.0 (https://github.com/TransDecoder (accessed on 16 October 2022)) based on expressed sequence tags obtained from the prediction above. For functional annotation, we compared all predicted genes to the NT database, NR database, the Swissprot database, and the KEGG database using BLAST v2.11 [41], diamond v2.0.9 [42], and Interproscan v5.52-86 software [43], respectively.

Finally, a circular genome visualization map was constructed by Circos v0.69-8 [44] software to show the results of genome assembly and genome annotation.

### 2.7. Comparative Genome Analysis

Phylogenetic analysis was conducted using three *Luffa* genomes (*L. acutangula SG261*, *L. acutangula* [10], and *L. cylindrica* [11]) and another 10 species of Cucurbitaceae (*Benincasa hispida* [45], *Citrullus lanatus* [46], *Cucumis melo* [47], *Cucumis sativus* [48], *Cucurbita argyrosperma* [49], *Cucurbita maxima* and *Cucurbita moschata* [50], *Cucurbita pepo* [51], *Lagenaria siceraria* [52], and *Momordica charantia* [53]). The data were downloaded from Cucurbit Genomics Database (http://cucurbitgenomics.org/ (accessed on 20 October 2022)) and NCBI (https://www.ncbi.nlm.nih.gov/ (accessed on 20 October 2022)). OrthoFinder v2.5.4 [54] was used to identify orthologous groups for these species. The protein sequences of all single-copy genes were identified, and multiple sequence alignment was performed by MAFFT v7.487 [55] and trimAI v5.7.5 [56]. RAxML v8.2.12 [57] was used to construct a phylogenetic tree for each group of single-copy proteins, and a coalescent-based phylogenetic tree was constructed using ASTRAL v5.7.5 [58].

The software minimap2 v2.21 was used to compare the SG261 assembly genome with other *Luffa* genomes (*L. acutangula* [10] and *L. cylindrica* [11]). The alignment results of mapq60 were retained, and the PAFR v0.0.2 software was used to visualize the alignment results and obtain the synteny dot plots.

### 2.8. Structural Variants Analysis

Short reads were used to identify SNP and INDEL between SG261 genome and *L. cylindrica* genome (downloaded from NCBI, SRR10818295). Short reads were mapped to the assembled genome using BWA v0.7.17 with parameters “-R ‘@ rg\tid: lae518\TPU: lae518\TSM: lae518’”. Sambamba v0.8.0 [59] was used to mark and filter PCR duplication, and then SNP and InDel were called using bcftools v1.13 software [60]. Finally, densities of SNPs and InDels were calculated with a window size of 400 kb and displayed on the Circos plot.

Long reads were used to identify structural variants (SVs) between the *L. acutangula SG261* genome and the *L. cylindrica* genome [11]. Minimap2 v2.21 was used to map the PacBio long reads of SG261 to the *L. cylindrica* genome with parameter “--MD -ax map-pb”, and samtools v1.13 [61] was used for sorting and indexing. Then, SVs were called using Sniffles v1.0.12 [62] with parameter “-l 50 -t 45”. The long reads of the *L. cylindrica* genome were downloaded from NCBI (SRP239503). Then, the long reads were mapped to SG261 genome, and SVs were called using the same method above.

We then filtered the SVs using the SG261 genome as a reference genome according to a previous study [13]. First, we removed imprecise SVs and SVs on scaffolds. Then, SVs longer than 100 kb in length and genotype of “0/0” were removed. Finally, we identified regions prone to false SVs and removed SVs that intersected with them. To identify these regions, PaSS [63] was used to simulate long reads based on the *L. acutangula* genome [10]. PacBio long reads of SG261 and long reads simulated by *L. acutangula* were mapped to the SG261 genome using minimap2, and SVs were called using Sniffles. SVs larger than 100 kb and imprecise SVs were then removed to obtain the final vcf file. Bedtools v2.30.0 [64] was used to extract the SVs between *L. acutangula SG261* and *L. cylindrica*, which were demonstrated to intersect with the 2 vcf files. In addition, according to the genome annotation file, the genes affected by SVs were identified with Bedtools.

We identified PAVs between *L. acutangula SG261* and the *L. cylindrica* genome using a sliding window method [17]. We divided the *L. cylindrica* genome into 500 bp windows with 100 bp as the step size and then mapped it with the assembled genome using BWA to identify PAV. According to the genome annotation file, the genes affected by PAVs were identified with Bedtools.

### 2.9. GO Enrichment and Functional Annotation of Genes with SVs and PAVs

Genes with SVs and PAVs were submitted to the NT database, NR database, and Swissprot database for functional annotation using BLASTN v2.11 and diamond v2.0.9 software, respectively. In addition, we used SG261 genome as the background and SVs and PAV-associated genes as foreground for GO enrichment. Interproscan v5.52-86 was used to annotate the SG261 genome genes with parameters “-f TSV -iprlookup -goterms -pa”. ClusterProfiler v4.0.0 [65] was used to enrich SV-related genes and PAV-related genes. *p* < 0.05 was a significant enrichment.

### 2.10. Analysis of Expansion and Contraction for Flowering-Time-Related Genes

According to the known flowering-related genes [66], we searched the annotation results of SV-related genes and found that there were 16 genes that might be related to flowering time. Then, we performed gene family expansion and contraction for some flowering-related gene families, including FT [67] and CO-like gene families [68], as well as unidentified FY (*FLOWERING LOCUS Y*) and EFM (EARLY FLOWERING MYB PROTEIN) families and some MYB [69], AP2/ERF [70], bZIP [71], NAC [72], and WAKY transcription factor families. We downloaded the sequences of these families in cucumber based on previous gene family analysis, and the unidentified FY and EFM were retrieved according to the NCBI database. The heatmap was obtained using TBtools [73].

## 3. Results and Discussion

### 3.1. Genome Sequencing and Assembly

A high-quality chromosome-level genome of the inbred line SG261 was assembled in the present study. Firstly, 74.96 Gb clean reads were obtained by the MGI-SEQ 2000 sequencing system (Table S1). Based on 17-mer frequency, the estimated genome size was 879.83 Mb with a heterozygosity rate of 0.49% and 73.00% repetitive sequences (Figure S1, Table S2). Secondly, a total of 142.75 Gb in PacBio clean reads was generated and used for contig assembly (Table S1). The assembled contig genome was composed of 283 contigs with a total length of 739.31 Mb, of which contig N50 was 18.38 Mb in length, with an average length of 2.61 Mb (Table 1 and Table S4). Thirdly, 70.24 Gb of Hi-C data was obtained by the BGI MGI-SEQ 2000 sequencing system for assembly (Table S1). Based on Hi-C data, the contig sequences were anchor-corrected to obtain a scaffold-level genome. The 283 contigs were anchored to 31 scaffolds, including 13 pseudo-chromosomes and 18 scaffolds, of which the shortest was 50.01 Mb (chr12) and the longest was 64.19 Mb (chr01) (Table S5). The final pseudo-chromosomal level genome had a total length of 739.82 Mb, and the scaffold N50 was 56.08 Mb (Table 1). Finally, the Hi-C data were mapped to the assembled genome, and the Hi-C correlation heatmap obtained by HiCExplorer showed that the Hi-C-assisted assembly was high quality (Figure S2).

We also aligned short reads to the assembled genome, and 99.51% of the data could be mapped to the assembled genome, indicating that the assembled genome contained most of the genome information. The completeness of the assembled genome was 91.25%, evaluated by BUSCO (Table S3), which indicated that the SG261 genome assembly was complete and could be used for subsequent analysis. Compared with other *Luffa* genomes [10,11,12], the assembled genome of SG261 had the highest N50. In addition, we found that the SG261 genome and another *L. acutangula* genome are larger than the three *L. cylindrica* genomes, which confirmed that the genome of *L. acutangula* is larger than that of *L. cylindrica* (Table 2).

### 3.2. Genome Annotation

**Figure 2:**
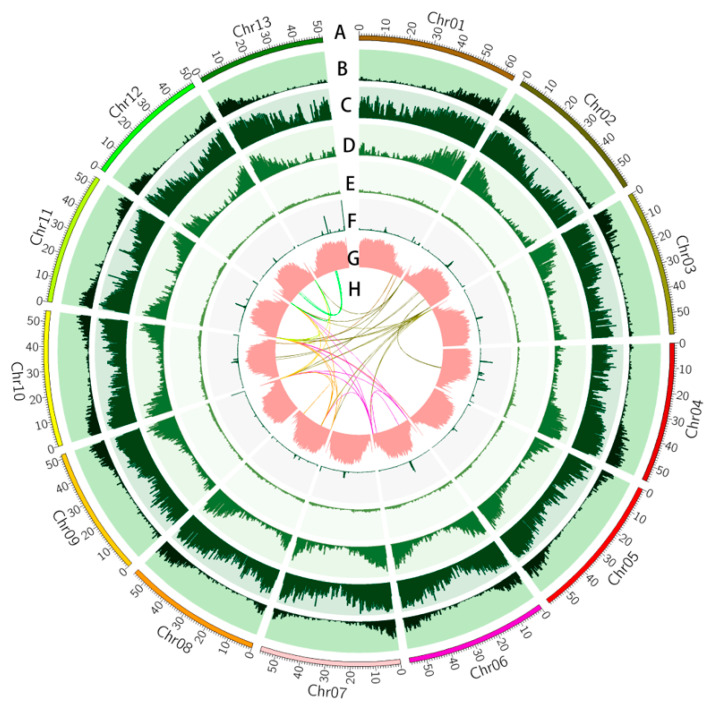
Circos graph of the *L. acutangula SG261* genome’s characteristics. A, physical map of 13 chromosomes (Mb scale); B, gene density, the number of genes in 400 kb windows; C, SNP density, the number of SNPs between *L. acutangula SG261* and *L. cylindrica* in 400 kb windows; D, InDel density, the number of InDels between *L. acutangula SG261* and *L. cylindrica* in 400 kb windows; E, DNA type transposon elements (DNA-TEs) density, the coverage of DNA-TEs in 400 kb windows; F, tandem repeat (TR) density, the coverage of TRs in 400 kb windows; G, long terminal repeat (LTR) density, the coverage of LTRs in 400 kb windows; H, syntenic blocks.

EDTA was used to annotate the complete repetitive sequences of the assembled genome, and RepeatMasker was used to annotate the incomplete repetitive sequences based on the results of EDTA. The results showed that 536.83 Mb (72.56% of the assembled genome) of the sequences was annotated as repetitive sequences, including 67.84% of the retrotransposon long terminal repeats (LTRs) and 4.72% of the DNA type transposon elements (Figure 2). Copia and Gypsy elements are the main elements in LTRs, 276,809 and 261,064 in number, covering 178.81 Mb and 227.38 Mb, accounting for 24.17% and 30.73% of the genome, respectively (Table S6). In addition, we also annotated the tandem repeats (TRs) using TRF, which are enriched at centromeric regions (Figure 2). The accumulation of repetitive elements, especially LTRs, may be the reason for the difference in k-mer size and assembly size of the genome [74] and also lead to the large genome of *Luffa* [10]. Comparing several *Luffa* genomes, it was found that although the genome of *L. acutangula* was generally larger than that of *L. cylindrica*, the length of non-repetitive sequences was less than that of *L. cylindrica* (Table S6). Therefore, a large number of repetitive sequences may lead to the larger genome of *Luffa* as compared to other Cucurbitaceae plants.

A total of 27,312 protein-coding genes were annotated in the assembled genome, with an average transcript length of 1305.67 bp (Table 2 and Table S6). Compared with other *Luffa* genomes [10,11,12], there are fewer protein-coding genes in SG261 than other *Luffa* genomes (Table 2 and Table S6). In addition, we functionally annotated all protein-coding genes, and a total of 25,356 genes were annotated, accounting for 92.84% of all protein-coding genes (Table S7).

### 3.3. Synteny and Phylogenetic Analysis

**Figure 3:**
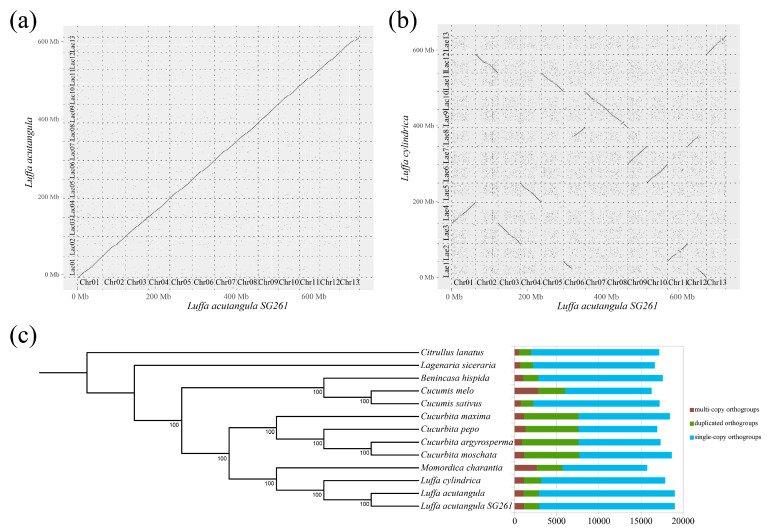
Comparative genome analysis of *L. acutangula SG261*. (**a**) Comparisons between the genomes of *L. acutangula SG261* and another *L. acutangula*. (**b**) Comparisons between the genomes of *L. acutangula SG261* and *L. cylindrica*. (**c**) Phylogenetic tree of *L. acutangula SG261* and other representative Cucurbitaceae genomes based on single-copy orthologous protein sequences. Bar charts display distribution of orthologous in *L. acutangula SG261* and 12 other sequenced Cucurbitaceae genomes, multiple copies (brown), two copies (green), and single copy (blue).

Synteny analysis indicated that the SG261 assembled genome showed good linearity with the published *L. acutangula* genome [10] (Figure 3a). The SG261 assembled genome showed relatively low linearity with the *L. cylindrica* genome [11] with many mismatches of chromosomes. Among them, chromosomes 1, 9, 10, 11, and 13 of SG261 corresponded to chromosomes 4, 7, 6, 2, and 13 of the *L. cylindrica* genome, respectively, with same genome directions; chromosomes 2, 3, 4, 5, 7, and 8 of SG261 corresponded to chromosomes 12, 3, 5, 11, 10, and 9 of the *L. cylindrica* genome, respectively, with some reverse complementary strands (Figure 3b). In addition, we also found that the SG261 assembled genome has chromosome translocation between chromosomes 6 and 12 (chromosomes 1 and 8 of *L. cylindrica*), and there were small segments of chromosome inversion in chromosomes 7, 8, and 11 of the SG261 genome (chromosomes 10, 9, and 2 of *L. cylindrica*) (Figure 3b).

Three *Luffa* genomes and 10 other Cucurbitaceae genomes were used for phylogenetic analysis, and the homologous gene clusters of 13 species were identified (Figure 3c). Through homologous gene cluster analysis, we found that the number of single-copy and two-copy genes in the three *Luffa* genomes were similar (Figure 3c, Table S8). Phylogenetic analysis indicated that the three *Luffa* genomes were clustered to a branch, and the evolution time of *L. acutangula* was later than that of *L. cylindrica*, which confirmed the close relationship of the three *Luffa* genomes. Bitter gourd (*M. charantia*) was the closest cucurbit vegetable to the *Luffa* species, followed by *Cucurbita*, *Cucumis*, *Benincasa*, *Lagenaria*, and *Citrullus*, which confirmed the results in the previous study [10,75].

### 3.4. Variation between Luffa acutangula SG261 and Luffa cylindrica

Generally, genomic variation can be mainly divided into single-nucleotide polymorphisms (SNPs), small insertion or deletions (InDels, ≤50 bp), and structural variations (SVs, >50 bp) [76]. To explore the differences between *L. acutangula* and *L. cylindrica*, we used short-read data and long-read data to identify variants. Taking the *L. acutangula SG261* genome as the reference genome, 11,505,354 variants were identified by the short reads mapped from the *L. cylindrica* genome, including 11,017,265 SNPs and 488,089 InDels, with an average of 14.89 SNPs and 0.66 InDels per kb. Interestingly, the density distribution of SNPs and InDels shared the same genomic regions with the gene density distribution (Figure 2). The density of SNPs and InDels was lower in the regions with dense repetitive elements but higher in the regions with high gene density. SNPs and InDels have the same density distribution in the genomes of pak choi, Chinese cabbage, and oilseed yellow sarson [17]. Most SNPs and InDels in the gene region come from the intergenic region, and the gene sequence is relatively conservative [77]. The high variation density of chromosome arm regions may be caused by the variation in intergenic regions.

**Figure 4:**
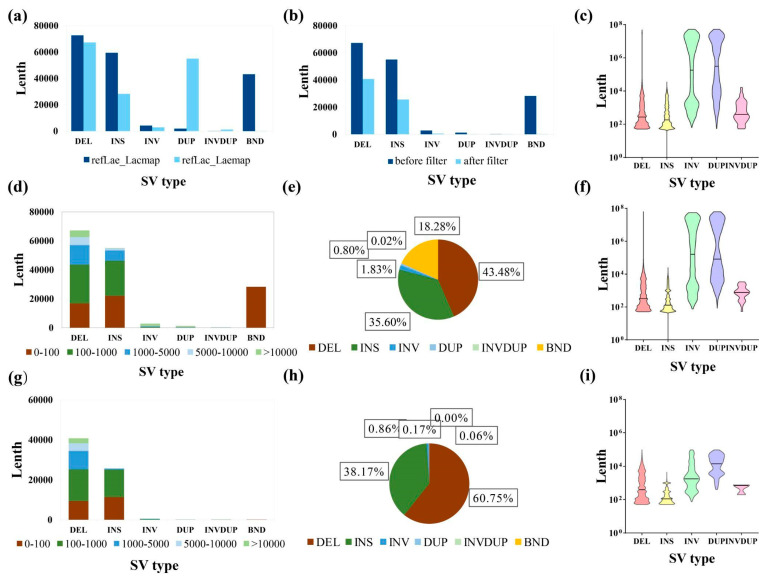
Statistics of structural variations (SVs) between *L. acutangula SG261* and *L. cylindrica*. (**a**) Statistical graph of the quantitative differences in different structural variation types between *L. acutangula SG261* as reference and *L. cylindrica* as reference. (**b**) Statistical graph of the quantitative difference in different structural variation types between before and after filtering detected with *L. acutangula SG261* as reference. (**c**) Violin plot of distribution law of different structural variation types detected with *L. cylindrica* as reference genome. (**d**) Bar chart of the proportion of different SV lengths in different structural variation types detected with *L. acutangula SG261* as reference genome. (**e**) Percent pie chart of different structural variation types detected with *L. acutangula SG261* as reference. (**f**) Violin plot of distribution law of different structural variation types detected with *L. acutangula SG261* as reference genome. (**g**) Bar chart of the proportion of different SV lengths in different structural variation types after filtering detected with *L. acutangula SG261* as reference genome. (**h**) Percent pie chart of different structural variation types after filtering detected with *L. acutangula SG261* as reference genome. (**i**) Violin plot of distribution law of different structural variation types after filtering detected with *L. acutangula* SG261 as reference genome.

Structural variations were identified using the PacBio long reads of *L. acutangula SG261* and *L. cylindrica*. Analysis of SVs between the genomes of SG261 and *L. cylindrica* produced 154,594 SVs identified with SG261 as the reference genome and 181,460 SVs identified with *L. cylindrica* as the reference genome. Among the different types of SVs, there are more DUP and INVDUP (complex chimeric variants with both inversions and duplications on the chromosome) SVs identified in *L. acutangula SG261* and more DEL, INS, INV, and BND SVs in *L. cylindrica* (Figure 4a, Tables S9 and S10). The length and number of different types of SVs and their distribution in the two genomes were the same (Figure 4c,f). We mainly focused on the SVs identified with *L. acutangula SG261* as the reference genome. A total of 67,128 SVs were obtained after filtering (Table S11). The DEL and INS were the main types of variation in SVs, accounting for 43.8% and 35.6% of the total SVs before filtering and 60.75% and 38.17% of the total SVs after filtering (Figure 4d–h). The lengths of DEL and INS variants were mostly between 100 and 1000 bp (Figure 4f–i). For INV and DUP variants, the SVs before filtering were mainly distributed in the two ranges (1000–5000 bp and >10,000 bp). The distribution of SVs after filtering was similar to that of before filtering (Figure 4d,f,g,i and Table S12). Finally, we extracted 2424 genes with SVs in CDS regions based on the genome annotation results and the SV data.

Presence/absence variations (PAVs) were important variant types that differed between *L. acutangula SG261* and *L. cylindrica*. We identified 55,978 PAVs in the *L. acutangula SG261* genome, covering 43.38 Mb bases with 1094 genes, and 67,178 PAVs were identified in the *L. cylindrica* genome, covering 56.37 Mb bases with 1108 genes (Table S13). Analysis of the length and number distribution of PAVs found that the PAVs with a length of 500 bp were the most abundant (Figure S3). The longest PAVs in SG261 and *L. cylindrica* were 53,600 bp and 28,900 bp, respectively (Table S13).

### 3.5. Functional Annotation and GO Enrichment of SV and PAV Genes

In order to explore whether structural variation affects the phenotypic difference between *L. acutangula* and *L. cylindrica*, we performed functional annotation and GO enrichment for 2424 SV genes and 1094 PAV genes. We aligned SV and PAV genes to NT, NR, and Swissprot databases for functional annotation. A total of 2054 genes can be annotated in the 2424 SV genes. A total of 870 genes can be annotated in the 1094 PAV genes.

**Figure 5:**
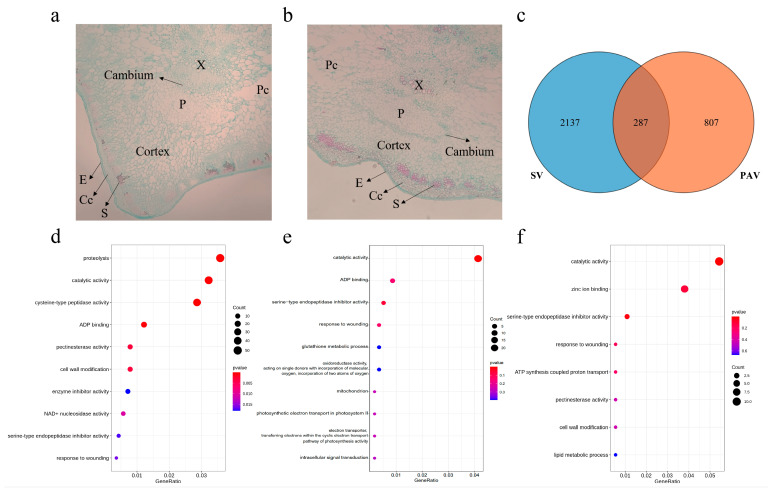
Section observation of *Luffa* angular slices and GO enrichment of SV and PAV genes in *L. acutangula SG261*. (**a**) Section observation of angular of *L. acutangula*. (**b**) Section observation of angular of *L. cylindrica*. (**c**) Venn diagram shows the overlap of genes between SV and PAV. (**d**) GO enrichment map of SV-related genes. (**e**) GO enrichment map of PAV-related genes. (**f**) GO enrichment map of the same genes for SV and PAV. E, epidermal; Cc, collenchymatous cell; S, sclerenchyma; P, phloem; X, xylem; Pc, parenchyma cell.

We carried out GO enrichment for 2424 SV genes, which were enriched for proteolysis (GO:0006508), catalytic activity (GO:0003824), cysteine-type peptidase activity (GO:0008234), ADP binding (GO:0043531), pectinesterase activity (GO:0030599), and cell wall modification (GO:0042545), as well as others (Figure 5d). The PAV genes were enriched for catalytic activity (GO:0003824), ADP binding (GO:0043531), serine-type endopeptidase inhibitor activity (GO:0004867), and response to wounding (GO:0009611), as well as others (Figure 5e). Subsequently, we obtained 287 genes containing both SVs and PAVs using TBtools (Figure 5c). These genes are enriched for catalytic activity (GO:0003824), zinc ion binding (GO:0008270), serine-type endopeptidase inhibitor activity (GO:0004867), response to wounding (GO:0009611), and pectinesterase activity (GO:0030599), among others (Figure 5f).

### 3.6. Structural Variation Genes Involved in Ridge Development

Ridges are the most important phenotype in *L. acutangula* in contrast to the absence of ridges in *L. cylindrica*. Compared with *L. cylindrica*, the cortical cells in *L. acutangula* grow faster longitudinally, resulting in longer and larger cortical cells. In addition, there are more cortical cells in *L. acutangula* than *L. cylindrica* at the ridge position (Figure 5a,b).

GO enrichment of SV- and PAV-related genes enriched for pectinesterase activity and cell wall modification can be seen in Figure 5c–f. For example, the DEL (49,297,508–49,311,124 bp) of chromosome 9 affected the pectinesterase PPME1-like gene (Lac_SG261_V1bChr09g017340.1) and the pectinesterase 63 gene (Lac_SG261_V1bChr09g017350.1); the DEL (50,615,779–50,616,960 bp) of chromosome 10 leads to the deletion of a large fragment of the pectinesterase/pectinesterase inhibitor 7 gene (Lac_SG261_V1bChr10g019340.1) and so on (Table S14). Pectin gels are capable of large changes in hydration and stiffness, which can alter the behavior of cells and tissues. For example, increasing the stiffness of cell wall pectin gel may result in decreased cell growth or if stiffened enough may cause cell–cell separation by gel fracture [78]. Variation in pectinesterase may lead to variation in cell growth, thus affecting the growth of fruits. Therefore, these genes affected by SVs and PAVs may play a role in the development of the ridges of *L. acutangula*.

### 3.7. Analysis of Flowering-Time-Related Genes

**Figure 6:**
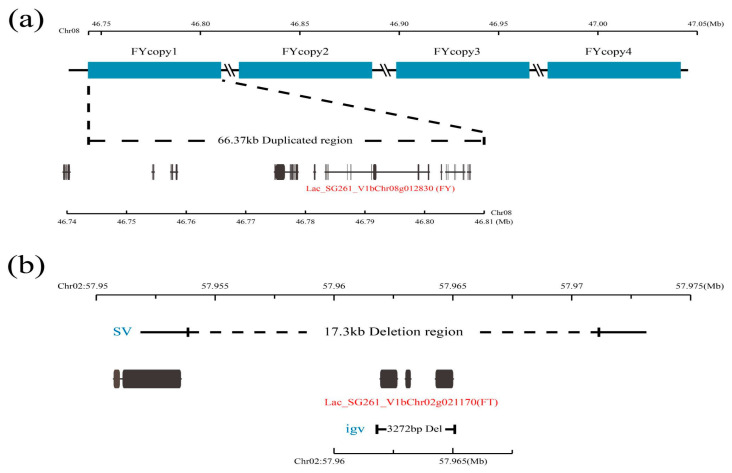
Schematic diagram of structural variation in major flowering-related genes. (**a**) Schematic diagram of DUP variation at the location of *FY* gene. (**b**) Schematic diagram of DEL variation at the location of *FT* gene.

Flowering is an important process that determines the success of plant reproduction. Plants must accurately combine internal and environmental signals to initiate the flowering process [66]. Flowering time has a very important influence on plant fitness and yield [79]. There are different flowering times between *L. acutangula* and *L. cylindrica*. The regulatory pathway of plant flowering has a relatively formed framework, and the flowering regulatory pathway map has been integrated in previous studies [66]. Compared to SNPs, SVs can cause large-scale perturbations of cis-regulatory regions and are therefore more likely to quantitatively change gene expression and phenotypes [13]. Based on the flowering regulatory pathway map and functional annotation of SV genes, we found 17 genes with SVs that may affect the flowering of *Luffa* (Table S15). These 17 genes are mainly affected by DEL-, INS-, and DUP-type SVs, among which a 78 bp insertion on chromosome 1 leads to an insertion of the *PIE1* (Lac_SG261_V1bChr01g011540.1) gene; a 17,284 bp DEL of SG261 on chromosome 2 leads to the deletion of three adjacent *FLOWERING LOCUS T* (*FT*) genes (Lac_SG261_V1bChr02g021160.1—Lac_SG261_V1bChr02g021180.1) (Figure 6b, Table S15), the *FT* receives feedback from the upstream regulatory mechanism and has great influence on flowering [80]; a DUP on chromosome 8 leads to four duplications of three *FLOWERING LOCUS Y* (*FY*) genes (Lac_SG261_V1bChr08g012820.1, Lac_SG261_V1bChr08g012830.1, and Lac_SG261_V1bChr08g012880.1) (Figure 6a, Table S15), and the *FY* gene mutant blooms later in both long-day and short-day conditions [81,82]. In addition, some transcription factors may affect flowering time (Table S15). These genes with SVs may affect the flowering time in *Luffa*.

**Figure 7:**
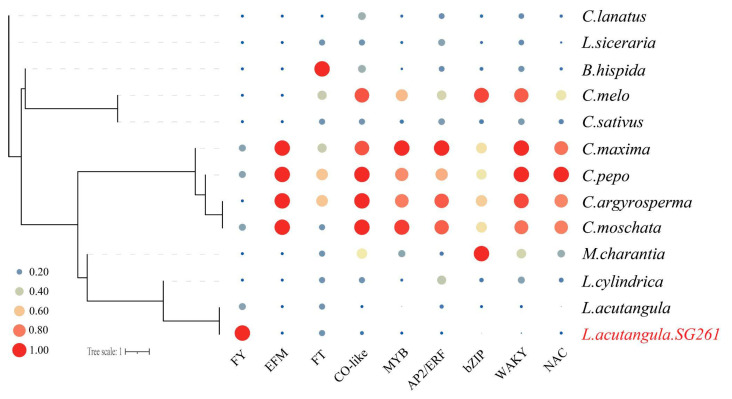
Expansion analysis of flowering-related gene families and some transcription factor gene families.

To explore the quantitative changes in flowering-related gene families in evolution, we performed expansion and contraction analysis of some gene families and genes. We downloaded these gene families and their protein sequences in cucumber, constructed homologous gene clusters of all genes based on protein sequences using Orthofinder v2.5.4, and checked the expansion and contraction of gene families according to the gene clusters (Figure 7). There are more *FY* genes in SG261 than other species, which further verified the authenticity of DUP variation at the position of *FY* genes. However, there was no significant difference in the number of genes in some flowering-related gene families, such as *CO-like*, *FT,* and *EFM* gene families, between SG261 and *L. cylindrica* (Table S16).

## 4. Conclusions

In this study, we reported a chromosome-scale reference genome assembly of *L. acutangula*. Our ribbed loofah genome enriches the loofah gene pool, and the sequenced varieties in this study are the best varieties selected and bred by our organization, which lays the data foundation for the subsequent research of our organization. In the structural variation analysis, the two ribbed loofah genomes allowed us to exclude the interference of intraspecific variation to a certain extent. The evolution time of *L. acutangula* was later than that of *L. cylindrica*, and there were some chromosomal translocations and large sequence inversions between *L. acutangula* and *L. cylindrica*. A total of 2424 and 1094 genes were affected by SVs and PAVs, respectively, in which 17 *FT* and *FY* genes may be related to the differences in flowering time observed in these *Luffa* species. This high-quality genome assembly and structural variation information will greatly facilitate the research of the molecular mechanisms of agronomic traits and provide valuable insights for pan-genome research and the molecular breeding of *Luffa*.

## Figures and Tables

**Table 1:** Summary for assembly of *L. acutangula SG261*.

Sample ID	Illumina + PacBio + Hi-C			
	Length		Number	
	Contig (bp)	Scaffold (bp)	Contig	Scaffold
Total	739,313,328	739,817,328	283	31
MAX	37,192,690	64,188,378	-	-
mean	2,612,414	23,865,075	-	-
A	236,049,024	236,049,024	31.93%	31.91%
C	133,750,610	133,750,610	18.09%	18.08%
T	235,878,785	235,878,785	31.91%	31.88%
G	133,634,909	133,634,909	18.08%	18.06%
N	-	504,000	-	0.07%
N50	18,375,156	56,077,004	15 (5.30%)	7 (22.58%)
N60	13,378,742	55,168,345	20 (7.07%)	8 (25.81%)
N70	10,763,832	54,241,644	26 (9.19%)	9 (29.03%)
N80	8,026,258	51,658,337	34 (12.01%)	11 (35.48%)
N90	4,503,403	51,095,587	46 (16.25%)	12 (38.71%)

**Table 2:** Comparison of several *Luffa* genomes.

	*Luffa * *cylindrica* (L.) [12]	*Luffa* *cylindrica* (L.) [11]	*Luffa* *Cylindrica* [10]	*Luffa acutangula* [10]	*Luffa* *acutangula* *SG261*
Sequence method	Pacbio	Pacbio	Pacbio	Pacbio	Pacbio
N50 (contig) (bp)	4,815,853	8,800,239	-	110,403	18,375,156
N50 (scaffold) (bp)	48,664,788	48,760,765	578,616	47,609,564	56,077,004
Genome size (Mb)	669.7	656.2	689.8	735.6	739.8
Longest scaffold (bp)	62,749,569	55,641,800	7,054,290	56,032,585	64,188,378
Repetitive sequence length (bp)	416,310,000	419,095,893	391,650,000	456,690,000	536,834,300
Non-repeated sequence length (bp)	253,398,411	237,094,093	298,222,192	278,920,612	202,983,028
BUSCO	91.6%	95.5%	93.00%	92.70%	91.25%
Total number of genes	31,661	27,154	43,828	32,233	27,312
Average transcript (mRNA) length (bp)	-	4184.44	-	1508.632356	1350.665312
Average CDS length (bp)	1246.02	1160.18	-	1067.034598	1047.6968
Average exon length (bp)	218.87	241.63	258,1	233.5	294.6388316
Average gene length (bp)	4387.94	4734.773861	2582	2866	3404.439707

## Data Availability

Genome assemblies, raw genome and transcriptome sequencing reads have been deposited in the National Center for Biotechnology Information BioProject database under the accession PRJNA1042866.

## Supplementary Materials

The following supporting information can be downloaded at: https://www.mdpi.com/article/10.3390/plants13131828/s1, Figure S1. Distribution of K-mer frequencies for survey data. k-mer = 17. Figure S2. Hi-C heatmap of the genome assembly. Figure S3. Distribution of PAV between Lac and Lcy. Lac was *L. acutangula SG261*, Lcy was *L. cylindrica*. Table S1. Summary of sequencing data. Table S2. Statistical of 17-kmer genome estimation. Table S3. Quality assessment of the assembled genome of *L. acutangula SG261* BUSCO. Table S4. Length details of genome assembly. Table S5. Chromosome length of the genome assembly of *L. acutangula SG261*. Table S6. Summary of repetitive sequences in the *L. acutangula SG261* genome. Table S7. Functional annotations of *L. acutangula SG261* mRNA genes. Table S8. Summary statistics of the gene families of 13 Cucurbitaceae plant species. Table S9. Statistical of SVs between *L. acutangula SG261* and *L. cylindrica* with *L. cylindrica* as reference genome. Table S10. Statistical of SVs between *L. acutangula SG261* and *L. cylindrica* with *L. acutangula SG261* as reference genome. Table S11. Quantity statistics for filtering details of SVs with *L. acutangula SG261* as reference genome. Table S12. Statistical of filtered SVs between *L. acutangula SG261* and *L. cylindrica* with *L. acutangula SG261* as reference genome. Table S13. Statistics for PAV analysis of *L. acutangular SG261* and *L. cylindrica* genome. Table S14. List of genes enriched for pectinesterase activity (GO:0030599) and cell wall modification (GO:0042545). Table S15. Summary statistics of flowering-related gene. Table S16. Statistics of gene expansion and contraction in some gene families.
