# Supplementary material for: Genome Assembly and Structural Variation Analysis of Luffa acutangula Provide Insights on Flowering Time and Ridge Development
Source: Plants (Basel). 2024 Jul 3;13(13):1828. doi: 10.3390/plants13131828 (PMC11243878; doi:10.3390/plants13131828)
Supplement: Supplementary file 1 [file plants-13-01828-s001.zip › plants-2939423-supplementary.pdf]

Supplemental Figures and Tables

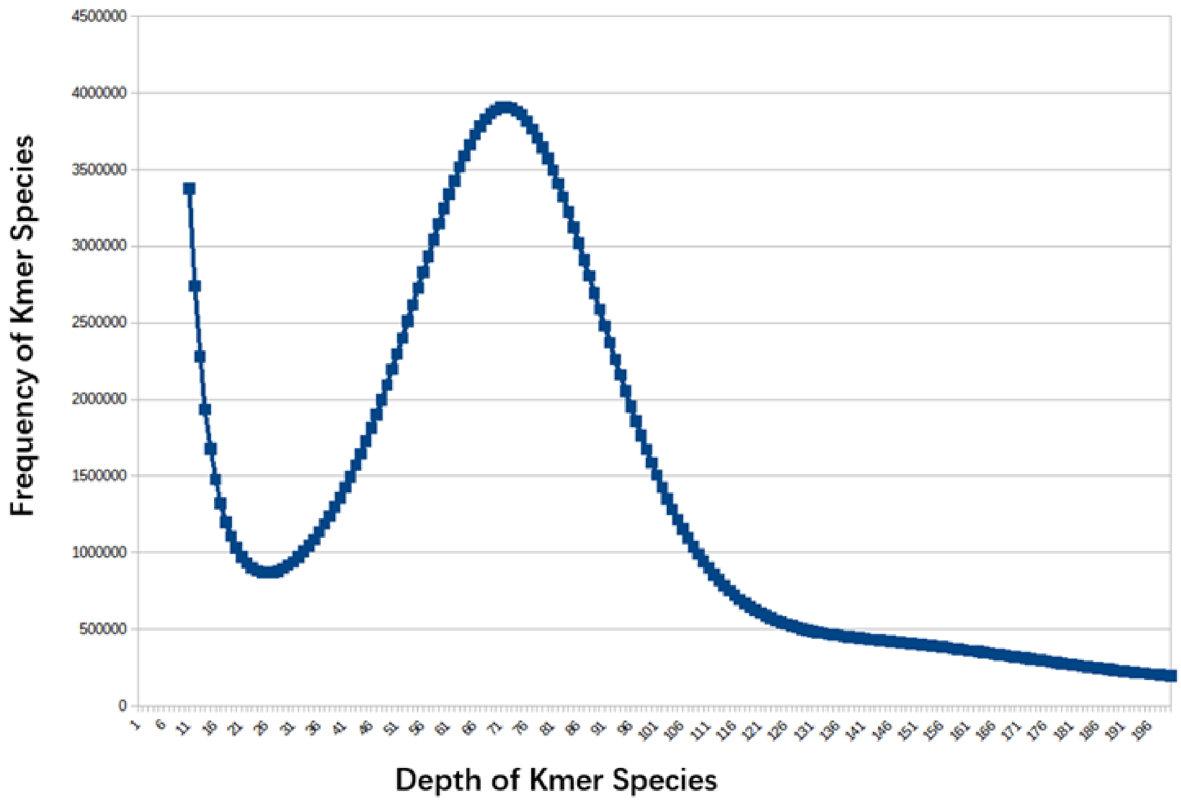


**Figure S1.** Distribution of K-mer frequencies for survey data. k-mer=17.

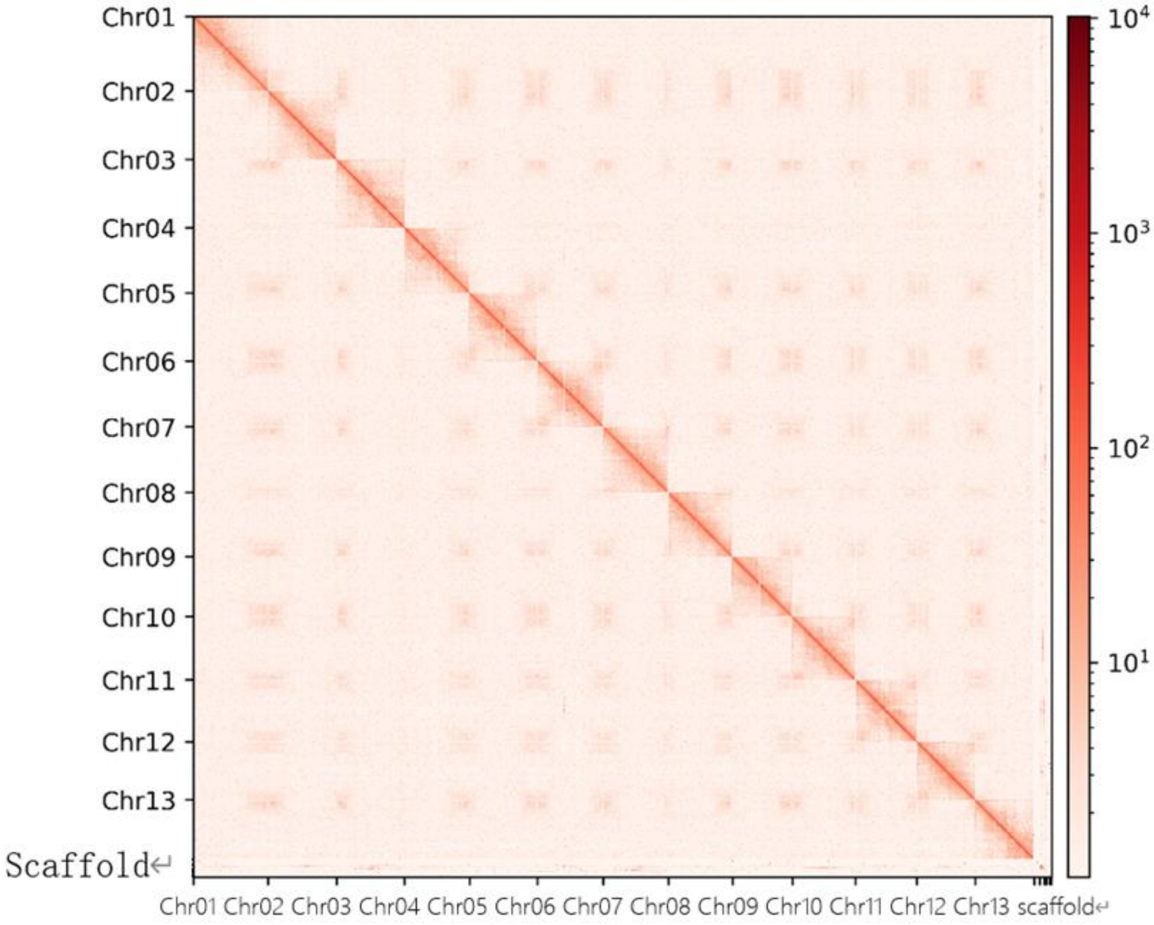


**Figure S2.** Hi-C heatmap of the genome assembly

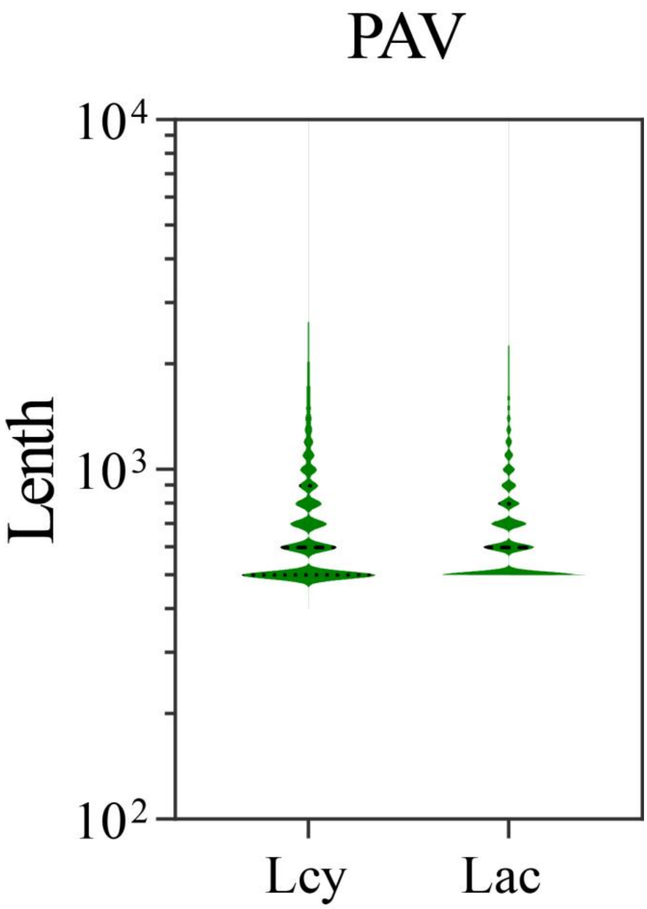


**Figure S3.** Distribution of PAV between Lac and Lcy. Lac was *L. acutangula SG261*, Lcy was *L. cylindrica*.

**Table S1.** Summary of sequencing data

| Sequencing platform | Read type | raw bases (Gb) | clean bases (Gb) | Insert size (bp) | Read length / subread N50 (bp) |
|---|---|---|---|---|---|
| MGI-SEQ 2000 | Illumina pair-end reads | 78.8 | 74.96 | 500 | 2 x 150 |
| MGI-SEQ 2000 | Hi-C pair-end reads | 70.24 | 70.24 | 500 | 2 x 150 |
| PacBio Sequel | PacBio subreads | 142.93 | 142.75 | - | 23284 |

**Table S2.** Statistical of 17-kmer genome estimation

| Genome Size | Heterozygous Ratio (%) | Repeat (%) |
|---|---|---|
| 879830000 | 0.49% | 73.00% |

**Table S3.** Quality assessment of the assembled genome of *L. acutangula SG261* BUSCO.

| Type | Number | Percent (%) |
|---|---|---|
| Complete BUSCOs (C) | 1,314 | 91.25% |
| Complete and single-copy BUSCOs (S) | 1,275 | 88.54% |
| Complete and duplicated BUSCOs (D) | 39 | 2.71% |
| Fragmented BUSCOs (F) | 25 | 1.74% |
| Missing BUSCOs (M) | 101 | 7.01% |
| Total BUSCO | 1,440 | 100.00% |

**Table S4.** Length details of genome assembly

| Reads statistics | contig_Length (bp) | contig_Number (%) | scaffold_Length (bp) | scaffold_Number (%) |
|---|---|---|---|---|
| N10 | 31,267,594 | 3 (1.06%) | 58,927,387 | 2 (6.45%) |
| N20 | 27,761,132 | 5 (1.77%) | 58,555,725 | 3 (9.68%) |
| N30 | 24,807,335 | 8 (2.83%) | 58,475,134 | 4 (12.90%) |
| N40 | 21,148,841 | 11 (3.89%) | 56,803,955 | 5 (16.13%) |
| N50 | 18,375,156 | 15 (5.30%) | 56,077,004 | 7 (22.58%) |
| N60 | 13,378,742 | 20 (7.07%) | 55,168,345 | 8 (25.81%) |
| N70 | 10,763,832 | 26 (9.19%) | 54,241,644 | 9 (29.03%) |
| N80 | 8,026,258 | 34 (12.01%) | 51,658,337 | 11 (35.48%) |
| N90 | 4,503,403 | 46 (16.25%) | 51,095,587 | 12 (38.71%) |
| Number | _ | 283 | _ | 31 |
| Mean lenth | 2,612,414 | _ | 23,865,075 | _ |
| Max length | 37,192,690 | _ | 64,188,378 | _ |
| Reads number | 739,313,328 | _ | 739,817,328 | _ |

**Table S5.** Chromosome length of the genome assembly of *L. acutangula SG261*.

| Chr | Size | Scaf Num |
|---|---|---|
| Chr01 | 64,188,378 | 1 |
| Chr02 | 58,927,387 | 1 |
| Chr03 | 58,555,725 | 1 |
| Chr04 | 56,077,004 | 1 |
| Chr05 | 58,475,134 | 1 |
| Chr06 | 56,803,955 | 1 |
| Chr07 | 56,198,377 | 1 |
| Chr08 | 55,168,345 | 1 |
| Chr09 | 51,658,337 | 1 |
| Chr10 | 54,241,644 | 1 |
| Chr11 | 53,190,697 | 1 |
| Chr12 | 50,014,713 | 1 |
| Chr13 | 51,095,587 | 1 |
| Unplaced | 15,222,045 | 18 |
| total | 739,817,328 | 31 |

**Table S6.** Summary of repetitive sequences in the *L. acutangula SG261*genome

| Types of repetitive sequences | Number | Length (bp) | Percentage of genome (%) |
|---|---|---|---|
| LTR | 798,122 | 501,925,758 | 67.84% |
| Gypsy | 276,809 | 178,814,990 | 24.17% |
| Copia | 261,064 | 227,380,203 | 30.73% |
| LTR_other | 260,249 | 95,730,565 | 12.94% |
| DNA TE | 151,511 | 34,908,542 | 4.72% |
| Total | 949,633 | 536,834,300 | 72.56% |

**Table S7.** Functional annotations of *L. acutangula SG261* mRNA genes.

| Values | Total | Nr | Nt | Swissprot | KEGG | Interpro | GO | Overall | unannotated |
|---|---|---|---|---|---|---|---|---|---|
| Number | 27,312 | 24,891 | 21,240 | 17,541 | 17,192 | 23,741 | 14,482 | 25,356 | 1,956 |
| Percentage | 100.00% | 91.14% | 77.77% | 64.22% | 62.95% | 86.93% | 53.02% | 92.84% | 7.16% |

**Table S8.** Summary statistics of the gene families of 13 Cucurbitaceae plant species.

| Species | multi-group | dup-group | single group | else counter |
|---|---|---|---|---|
| *Citrullus lanatus* | 556 | 1,399 | 15,227 | 16,626 |
| *Lagenaria siceraria* | 685 | 1,519 | 14,457 | 15,976 |
| *Benincasa hispida* | 1,050 | 1,775 | 14,786 | 16,561 |
| *Cucumis melo* | 2,845 | 3,184 | 10,270 | 13,454 |
| *Cucumis sativus* | 765 | 1,514 | 14,944 | 16,458 |
| *Cucurbita maxima* | 1,183 | 6,431 | 10,865 | 17,296 |
| *Cucurbita pepo* | 1,327 | 6,289 | 9,330 | 15,619 |
| *Cucurbita argyrosperma* | 909 | 6,668 | 9,776 | 16,444 |
| *Cucurbita moschata* | 1,138 | 6,555 | 10,986 | 17,541 |
| *Momordica charantia* | 2,630 | 3,077 | 10,066 | 13,143 |
| *Luffa cylindrica* (L.) | 1,177 | 1,972 | 14,776 | 16,748 |
| *Luffa acutangula* | 1,123 | 1,815 | 16,104 | 17,919 |
| *Luffa acutangula SG261* | 1,175 | 1,803 | 16,070 | 17,873 |

**Table S9.** Statistical of SVs between *L. acutangula SG261* and *L. cylindrica* with *L. cylindrica* as reference genome.

| Lenth (bp) \ SV type | **DEL** | **INS** | **INV** | **DUP** | **INVDUP** | **BND** | **other** | Total |
|---|---|---|---|---|---|---|---|---|
| 0-100 | 19657 | 20101 | 10 | 11 | 13 | 43201 | 0 | 82993 |
| 100-1000 | 31435 | 25062 | 757 | 145 | 58 | 0 | 0 | 57457 |
| 1000-5000 | 12928 | 10524 | 862 | 222 | 28 | 0 | 0 | 24564 |
| 5000-10000 | 4399 | 2916 | 108 | 126 | 5 | 0 | 1 | 7555 |
| 10000 | 4272 | 790 | 2466 | 1360 | 3 | 0 | 0 | 8891 |
| Total | 72691 | 59393 | 4203 | 1864 | 107 | 43201 | 1 | 181460 |

**Table S10.** Statistical of SVs between *L. acutangula SG261* and *L. cylindrica* with *L. acutangula SG261* as reference genome.

| Lenth (bp) \ SV type | **DEL** | **INS** | **INV** | **DUP** | **INVDUP** | **BND** | **other** | Total |
|---|---|---|---|---|---|---|---|---|
| 0-100 | 16851 | 22083 | 9 | 1 | 1 | 28260 | 1 | 67206 |
| 100-1000 | 26830 | 24166 | 479 | 67 | 18 | 0 | 0 | 51560 |
| 1000-5000 | 13479 | 7133 | 489 | 210 | 8 | 0 | 0 | 21319 |
| 5000-10000 | 5373 | 1447 | 80 | 91 | 0 | 0 | 0 | 6991 |
| >10000 | 4677 | 206 | 1765 | 870 | 0 | 0 | 0 | 7518 |
| Total | 67210 | 55035 | 2822 | 1239 | 27 | 28260 | 1 | 154594 |

**Table S11.** Quantity statistics for filtering details of SVs with *L. acutangula SG261* as reference genome.

| Filter type | before filter | filter | after filter |
|---|---|---|---|
| scaffold | 154,594 | 539 | 154,055 |
| imprecise | 154,055 | 39,119 | 114,936 |
| >100 kb | 114,936 | 2,281 | 112,655 |
| GT “0/0” | 112,655 | 35,697 | 76,958 |
| self SVs | 76,958 | 945 | 76,013 |
| BND >100 kb | 76,013 | 8,885 | 67,128 |

**Table S12.** Statistical of filtered SVs between *L. acutangula SG261* and *L. cylindrica* with *L. acutangula SG261* as reference genome.

| Lenth (bp) \ SV type | **DEL** | **INS** | **INV** | **DUP** | **INVDUP** | **BND** | **other** | Total |
|---|---|---|---|---|---|---|---|---|
| 0-100 | 9356 | 11340 | 3 | 0 | 0 | 38 | 0 | 20737 |
| 100-1000 | 15946 | 13809 | 199 | 6 | 3 | 0 | 0 | 29963 |
| 1000-5000 | 9191 | 471 | 214 | 35 | 0 | 0 | 0 | 9911 |
| 5000-10000 | 3805 | 0 | 42 | 8 | 0 | 0 | 0 | 3855 |
| >10000 | 2480 | 0 | 117 | 65 | 0 | 0 | 0 | 2662 |
| Total | 40778 | 25620 | 575 | 114 | 3 | 38 | 0 | 67128 |

**Table S13.** Statistics for PAV analysis of *L. acutangular SG261* and *L. cylindrica* genome

| | ***L. acutangular SG261*** | ***L. cylindrica*** |
|---|---|---|
| number of specific region | 55,978 | 67,178 |
| total length of specific region (bp) | 43,383,183 | 56,374,010 |
| length of longest specific region (bp) | 53,600 | 28,900 |
| location of longest specific region | HiC-scaffold 7: 101-53,700 bp | CM029395.1: 5,427,101-5,456,000 bp |
| number of specific gene | 1,094 | 1,108 |
| number of specific region longer than 5 kb | 148 | 210 |

**Table S14.** List of genes enriched for pectinesterase activity (GO:0030599) and cell wall modification (GO:0042545)

| gene ID | anntation | SVtype | SVstart | SVend | SVlen |
|---|---|---|---|---|---|
| Lac_SG261_V1bChr09g017340.1 | pectinesterase PPME1-like | DEL | 49297509 | 49311125 | -13616 |
| Lac_SG261_V1bChr09g017350.1 | pectinesterase 63 | DEL | 49297509 | 49311125 | -13616 |
| Lac_SG261_V1bChr10g004050.1 | pectinesterase/pectinesterase inhibitor 7 | INV | 3999780 | 4024856 | 25076 |
| Lac_SG261_V1bChr10g004090.1 | pectinesterase/pectinesterase inhibitor 7 | INV | 4003551 | 4086955 | 83404 |
| Lac_SG261_V1bChr10g004110.1 | pectinesterase/pectinesterase inhibitor 7 | INV | 4003551 | 4086955 | 83404 |
| Lac_SG261_V1bChr10g004130.1 | pectinesterase/pectinesterase inhibitor 7 | INV | 4003551 | 4086955 | 83404 |
| Lac_SG261_V1bChr10g004150.1 | pectinesterase/pectinesterase inhibitor 7 | INV | 4003551 | 4086955 | 83404 |
| Lac_SG261_V1bChr10g004170.1 | pectinesterase 2-like | INV | 4003551 | 4086955 | 83404 |
| Lac_SG261_V1bChr10g019340.1 | pectinesterase/pectinesterase inhibitor 7 | DEL | 50615780 | 50616961 | -1181 |
| Lac_SG261_V1bChr11g007160.1 | pectinesterase/pectinesterase inhibitor 21 | INV | 10225053 | 10237997 | 12944 |
| Lac_SG261_V1bChr12g011540.1 | pectinesterase/pectinesterase inhibitor 39 | DEL | 39780711 | 39825009 | -44298 |

**Table S15.** Summary statistics of flowering-related gene

| GeneID | annotation | chrom | Start (bp) | End (bp) | gene lenth (bp) | cds lenth (bp) | SVtype | SVstart (bp) | SVend (bp) | SVlen (bp) |
|---|---|---|---|---|---|---|---|---|---|---|
| Lac_SG261_V1bChr01g011540.1 | PIE1 | Chr01 | 50328894 | 50332611 | 3718 | 258 | INS | 50331846 | 50331846 | 78 |
| Lac_SG261_V1bChr02g021160.1 | FT | Chr02 | 57961941 | 57962672 | 732 | 732 | DEL | 57954211 | 57971495 | -17284 |
| Lac_SG261_V1bChr02g021170.1 | FT | Chr02 | 57963008 | 57963232 | 225 | 225 | DEL | 57954211 | 57971495 | -17284 |
| Lac_SG261_V1bChr02g021180.1 | FT | Chr02 | 57964269 | 57965018 | 750 | 750 | DEL | 57954211 | 57971495 | -17284 |
| Lac_SG261_V1bChr08g012820.1 | FY | Chr08 | 46781438 | 46781692 | 255 | 255 | DUP | 46743620 | 46809989 | 66369 |
| Lac_SG261_V1bChr08g012830.1 | FY | Chr08 | 46783332 | 46800771 | 17440 | 1377 | DUP | 46743620 | 46809989 | 66369 |
| Lac_SG261_V1bChr08g013080.1 | FY | Chr08 | 47021910 | 47053839 | 31930 | 2115 | DUP | 46968314 | 47038682 | 70368 |
| Lac_SG261_V1bChr09g003520.1 | VRN1/AP2 | Chr09 | 15735471 | 15736229 | 759 | 588 | INV | 15726880 | 15747962 | 21082 |
| Lac_SG261_V1bChr10g008020.1 | AGL29 | Chr10 | 8625375 | 8626004 | 630 | 630 | DEL | 8598410 | 8664599 | -66189 |
| Lac_SG261_V1bChr02g013950.1 | MYB | Chr02 | 16813819 | 16814818 | 1000 | 918 | DEL | 16789408 | 16815803 | -26395 |
| Lac_SG261_V1bChr03g011990.1 | MYB | Chr03 | 13571918 | 13572442 | 525 | 525 | DEL | 13570609 | 13574601 | -3992 |
| Lac_SG261_V1bChr07g012320.1 | MYB | Chr07 | 23865043 | 23867869 | 2827 | 1626 | INS | 23867174 | 23867177 | 141 |
| Lac_SG261_V1bChr09g004200.1 | MYB | Chr09 | 21507842 | 21508219 | 378 | 378 | DEL | 21506407 | 21512110 | -5703 |
| Lac_SG261_V1bChr10g000150.1 | MYB | Chr10 | 293815 | 294360 | 546 | 423 | INS | 294239 | 294240 | 83 |
| Lac_SG261_V1bChr04g013970.1 | HHO5/EFM | Chr04 | 49621013 | 49621398 | 386 | 303 | DEL | 49616642 | 49621727 | -5085 |
| Lac_SG261_V1bChr05g011140.1 | HHO6/EFM | Chr05 | 46708422 | 46709766 | 1345 | 981 | DEL | 46703153 | 46714953 | -11800 |
| Lac_SG261_V1bChr07g017180.1 | HHO5/EFM | Chr07 | 53062021 | 53062394 | 374 | 291 | DEL | 53061467 | 53070016 | -8549 |

**Table S16.** Statistics of gene expansion and contraction in some gene families

| Family ID | *Citrullus lanatus* | *Lagenaria siceraria* | *Benincasa hispida* | *Cucumis melo* | *Cucumis sativus* | *Cucurbita maxima* | *Cucurbita pepo* | *Cucurbita argyrosperma* | *Cucurbita moschata* | *Momodica charantia* | *Luffa cylindrica* | *Luffa acutangula* | *Luffa acutangula SG261* |
|---|---|---|---|---|---|---|---|---|---|---|---|---|---|
| FY | 1 | 1 | 1 | 1 | 1 | 2 | 2 | 1 | 2 | 1 | 1 | 2 | 5 |
| EFM | 2 | 2 | 2 | 2 | 2 | 4 | 4 | 4 | 4 | 2 | 2 | 2 | 2 |
| FT | 4 | 5 | 9 | 6 | 5 | 6 | 7 | 7 | 5 | 5 | 5 | 5 | 5 |
| CO-like | 12 | 11 | 12 | 18 | 11 | 18 | 19 | 19 | 19 | 14 | 11 | 9 | 10 |
| MYB | 117 | 117 | 116 | 158 | 120 | 185 | 167 | 170 | 182 | 131 | 117 | 113 | 112 |
| AP2/ERF | 134 | 140 | 134 | 156 | 138 | 209 | 178 | 198 | 197 | 127 | 152 | 127 | 117 |
| bZIP | 61 | 60 | 62 | 124 | 65 | 94 | 91 | 98 | 94 | 129 | 64 | 55 | 56 |
| WAKY | 60 | 60 | 61 | 88 | 62 | 93 | 93 | 90 | 86 | 70 | 63 | 53 | 54 |
| NAC | 78 | 76 | 78 | 105 | 83 | 127 | 139 | 124 | 125 | 92 | 82 | 74 | 73 |
